# Hyperacute vestibular syndrome: the role of an acute vertigo service

**DOI:** 10.3389/fstro.2023.1265009

**Published:** 2023-08-24

**Authors:** William Bierrum, Salman Haider, Michelle Balaratnam, Ali Alim-Marvasti, Arvind Chandratheva, Robert Simister, Nehzat Koohi, Diego Kaski

**Affiliations:** ^1^Comprehensive Stroke Service, National Hospital for Neurology and Neurosurgery, London, United Kingdom; ^2^Department of Brain Repair and Rehabilitation, Institute of Neurology, University College London, London, United Kingdom; ^3^Stroke Research Centre, UCL Queen Square Institute of Neurology, University College London, London, United Kingdom; ^4^Department of Clinical and Movement Neurosciences, Institute of Neurology, University College London, London, United Kingdom

**Keywords:** acute vestibular syndrome, vertigo, neuro-otology, vestibular, hearing loss, ataxia, stroke

## Abstract

Differentiating between peripheral and central causes of acute vertigo remains a clinical obstacle in the acute setting. Despite the availability of several validated diagnostic algorithms adoption and implementation of these tools is low because most emergency physicians are unfamiliar with them. Embedding an acute vertigo service within the emergency setting may help improve the diagnostic workup of patients presenting with this specific symptomatology and may have significant economic benefits, such as the avoidance of hospital admissions, reduction in unnecessary investigations, and decrease in length of hospital stay. In this work, we present four patients who were referred to the acute vertigo service at University College London Hospital (UCLH) for review. We discuss the indications for and limitations of implementing such a service.

## 1. Introduction

Acute vestibular syndrome (AVS) refers to rapid-onset, persistent vertigo or dizziness associated with nausea or vomiting, head-motion intolerance, gait instability, and the presence of spontaneous nystagmus (Hotson and Baloh, 1998). This is due to pathologies affecting the peripheral or central pathways. AVS accounts for ~12% of all neurological presentations to the emergency department (Royl et al., 2010). The most common cause of AVS is peripheral vestibular dysfunction (PVD), however, approximately 5% of all presentations are due to acute posterior circulation stroke (Cutfield et al., 2011). Vascular vertigo is typically acute and may be prolonged (≥24 h) or transient (<24 h) (Kim et al., 2022).

Differentiating between peripheral (typically benign, e.g., PVD) and central (potentially malignant, e.g., stroke) causes of AVS is particularly challenging in the hyperacute stage (within 24 h of onset), when rapid decisions surrounding treatment, evaluation, and hospital discharge are required (Kaski et al., 2023). The occasional negative neuroimaging in patients with acute isolated vascular vertigo (without other localizing symptoms) highlights the importance of appropriate bedside evaluation of patients presenting with AVS (Choi et al., 2018).

Clinical tools, such as the HINTS-plus (Head Impulse, Nystagmus, Test of Skew, plus Test of Hearing) algorithm, have been shown to differentiate peripheral from central AVS (Newman-Toker et al., 2015). However, their utility varies depending on the clinician's experience and training. A systematic review by Ohle et al. (2020) suggested that the HINTS examination, when used in isolation by Emergency Department (ED) clinicians, is not sufficiently accurate to rule out a central cause in patients presenting with AVS. In addition, this test is frequently performed in patients for whom the test has not been validated (e.g., in the absence of nystagmus) (Dmitriew et al., 2021).

Other diagnostic algorithms, namely TITRATE (Timing, Triggers, and Targeted bedside eye examinations), and STANDING (Spontaneous and positional nystagmus, evaluation of nystagmus direction, head impulse test, and evaluation of equilibrium), have been developed to help identify stroke as the underlying cause of acute vertigo. The diagnostic utility of these tests in the hands of emergency physicians (Nakatsuka and Molloy, 2022), who would benefit from such tools the most, is currently being evaluated.

Our recent survey of UK frontline clinicians suggests a lack of awareness and low confidence in the performance and interpretation of these diagnostic algorithms in UK emergency settings (Warner et al., 2022) and had an adoption rate of 9% following a 2-month implementation program (Rau et al., 2020). These algorithms also fail to highlight the importance of screening for central neurological features (e.g., facial weakness/sensory loss, diplopia, dysarthria, etc.) before focusing on nystagmus, if present.

Emergency physicians cannot easily achieve the same clinical skills as specialists. Instead, an important role is to appropriately triage patients who require specialist input. Acute vertigo is a diagnostically complex symptom with causes that may be otologic, neurologic, cardiovascular, or general medical. As such, appropriate triaging is a challenge in itself. Due to the prevalence of dizziness in the acute setting and the challenges for non-specialists in differentiating central from peripheral causes, many patients undergo inappropriate imaging, and a high proportion of those with strokes are missed (Saber Tehrani et al., 2018; Rau et al., 2020; Agarwal et al., 2022).

Several measures can be taken to help improve the diagnostic workup of patients presenting with acute neurological issues such as vertigo, which include neurological input at the start of the patient pathway through acute neurology services (NHS, 2021). This may include EDs and acute medical units within reach, in addition to acute neurology clinics. This can have significant benefits, namely the avoidance of admission and a reduced length of hospital stay (Moodley et al., 2019).

Such input can play a vital role in the early differentiation of benign conditions from dangerous disorders (Newman-Toker and Perry, 2015). In the absence of this provision, this role is usually filled by stroke physicians, but peripheral vestibular disorders again comprise the largest cohort of stroke mimics (24%) (Pohl et al., 2021). This may impact several outcome measures, namely thrombolysis accuracy and patient throughput, in both hyperacute inpatient and outpatient settings, e.g., TIA clinics.

We propose that an embedded acute vertigo service is one approach to meeting this need. In this paper, we discuss a series of patients referred to the acute vertigo service, consisting of a neurologist with an interest in vestibular neurology and a senior audiological scientist embedded within the acute neurovascular service at the University College London Hospital (UCLH), for review and discuss the indications and limitations for implementing such a service.

## 2. Patient summaries from the acute vertigo clinic

### 2.1. Patient 1

A 72-year-old man presented to the hyperacute stroke unit (HASU) with an acute onset of unsteady gait, a sensation similar to “feeling drunk,” and subjective dizziness. He reported slurred speech. His symptoms had deteriorated acutely in the preceding days before he presented to the hospital, but he had had a constant internal sensation of dizziness and unsteadiness for several months. He had a history of a middle cerebral artery (MCA) territory infarction in 2019 and a cerebellar infarction in 2022, both of which were embolic in origin and secondary to atrial fibrillation. Of note, he had presented with several episodes of similar symptoms without evidence of new infarctions on his magnetic resonance imaging (MRI), which were considered to be stroke “decompensations”. The patient was on warfarin for atrial fibrillation with a subtherapeutic international normalized ratio (INR) at presentation (1.7). An acute computed tomography (CT) scan, a CT angiogram performed 9 h after symptom onset, and a brain MRI the following day did not identify any new abnormalities. The head thrust test was reported as “positive” bilaterally. The patient was referred to the acute vertigo service for further review.

A specialist examination on the same day revealed a broad-based, unsteady gait with erratic foot placement and a tendency to reach for objects around the patient for support. The appearance was in keeping with a functional gait disorder. The shoulder tap test was positive, suggesting a functional gait disorder (Coebergh et al., 2021). There was no nystagmus, the vestibulo-ocular reflex (VOR) was deemed normal (confirmed using a video head impulse test), and there was no skew deviation. There was no positional nystagmus.

The above findings were not suggestive of a further vascular event or decompensation of the previous infarction. Following the acute vertigo review, the patient was discharged with a plan for referral to neuro-physiotherapy if the perceptual unsteadiness persisted.

### 2.2. Patient 2

A 92-year-old man presented to his local ED with a sudden onset of vomiting without dizziness or unsteadiness. Initially, he did not notice any hearing loss, tinnitus, oscillopsia, diplopia, dysarthria, or any limb weakness. He was diagnosed with possible benign paroxysmal positional vertigo (BPPV), although a Dix-Hallpike was not performed. He was given betahistine and discharged. Shortly after his discharge, he noticed imbalance and unsteadiness. He was unable to walk unassisted. He had three falls at home after his initial discharge and presented to another emergency department on two separate occasions, four and nine days after the initial episode. On evaluation in the ED, his examination did not show any apparent abnormalities, and a CT scan performed on the third visit showed an established left basal ganglia and right-sided pontine infarction. His past medical history included pre-diabetes and hypothyroidism, for which he was taking levothyroxine.

He was seen by the acute neurology team, which identified an unsteady gait with veering to the right. A left-beat nystagmus on the left lateral gaze was documented, along with a restricted upgaze and an abnormal Unterberger test (turning to the right). The head thrust test was abnormal to the right. The symptoms were attributed to a peripheral etiology, however, a central cause could not be excluded with certainty. As a result, acute vertigo service input was requested for further review.

On specialist acute vertigo review, he had impaired smooth pursuit, slow saccades, and bidirectional gaze-evoked nystagmus (more noticeable on right gaze, subtle on left gaze). He also had non-sustained upbeat nystagmus on his right gaze. The head impulse test revealed bilateral compensatory saccades (the VOR was more impaired on the left side on the formal vHIT). The test of skew did not reveal any apparent abnormalities. His gait was unsteady, but he was able to walk unaided.

His oculomotor tests, performed using video goggles, highlighted gaze-evoked nystagmus, slow saccades, and impaired smooth pursuit.

Although the borderline low VOR gain and compensatory saccades on the left suggested a peripheral vestibulopathy, the presence of central oculomotor signs (direction-changing nystagmus, impaired smooth pursuit, upbeat nystagmus) was not compatible with a peripheral cause. An MRI brain scan was performed and revealed an acute infarction in the right hemipons. The patient was admitted to the stroke unit for therapy input and treated with dual antiplatelet therapy for 3 months. His balance improved, and he was able to maintain independent living.

### 2.3. Patient 3

A 60-year-old woman presented to the UCLH stroke service with the acute onset of brief episodes of rotational vertigo, vomiting, imbalance, and oscillopsia occurring post-coitally. This was followed by further episodes of rotational vertigo on head motion, typically on sitting up, which tended to settle when lying flat. The patient had a history of hypertension, migraines, and severe motion sensitivity. On examination, she had a cautious gait but was able to stand unaided. Her eye movements were reported to be normal, but positional nystagmus was noted on Dix-Hallpike maneuvers. A presumptive diagnosis of bilateral BPPV was made.

The patient's initial head CT and CT angiogram did not show any signs of acute infarction. She was referred to the acute vertigo service for evaluation of the possibility of transient vascular vertigo (Kim et al., 2022).

A specialist's examination on the same day revealed no spontaneous or gaze-evoked nystagmus. Pursuit and saccadic eye movements were normal. Positional testing to the right evoked a right torsional nystagmus followed by a downbeat nystagmus of long duration (>60s). Left ear down elicited only downbeat nystagmus without fatigue and no crescendo-decrescendo pattern. Considering her symptoms started post-coitally, the patient underwent brain MRI and MRA scans (within 8 h of onset), which did not show an acute infarct or vascular abnormalities. She was given a working diagnosis of vestibular migraine and discharged on the same day with outpatient neuro-otology follow-up.

The delayed MRI (11 days later) and two visits to the outpatient neuro-otology clinic (2 weeks and several months later) did not find any new abnormalities. She was given pragmatic suggestions to help reduce her visual reliance and was referred for vestibular rehabilitation. She made a good recovery, had no further admissions due to her symptoms, and experienced infrequent and non-disabling episodes of vertigo related to impaired sleep and stress. The frequency of these episodes did not warrant preventative treatment but confirmed the diagnosis of vestibular migraine.

### 2.4. Patient 4

A 79-year-old man presented to the ED with dizziness and nausea. This was associated with a headache and unsteadiness that had been present for some time and were acutely worsening. The patient had seen his general practitioner (GP) that morning, who advised him to go to the ED immediately due to his unsteady gait. His symptoms improved by the time he arrived at the ED, and his neurological examination was reported as normal. To rule out a central cause of his symptoms, a comprehensive diagnostic work-up with blood tests, CT head, CT angiogram, and electrocardiogram (ECG) was performed, which did not show any acute neurological or cardiovascular causes. The patient was discharged and referred to the acute vertigo service for review.

A specialist's examination in the clinic 9 days later was normal from a neuro-otologic perspective. However, the Dix-Hallpike test revealed a clear right-sided posterior canal BPPV. The subject was treated with a Semont maneuver. He did not undergo any further evaluation for his acute dizziness after the specialist's visit and has remained asymptomatic.

## 3. Discussion

### 3.1. Learning points

The patient summaries highlight the range of underlying diagnoses in patients with acute vertigo. In each instance, a specialist review was central to the diagnosis. The availability of an acute vertigo service helped ensure the patient's care progressed correctly.

The acute vertigo service allows patients to receive an expert opinion in the hyperacute or acute setting, rather than months after the initial event. This was beneficial as it enabled prompt diagnosis, prevented unnecessary testing, and shortened the patient's length of stay.

For the specialist, assessing patients acutely helps to make a confident diagnosis, as it enables the identification of objective and sometimes subtle examination findings. Patient 1 had a history of previous strokes involving both the anterior and posterior circulation, in addition to prior episodes of suspected decompensation. The specialist review identified a functional gait disorder, which is recognized as a challenging diagnosis to make when it occurs in isolation (Nonnekes et al., 2020; Issak et al., 2023). In Patient 3, the specialist's evaluation identified vestibular migraine, an alternative diagnosis with a completely different treatment approach than BPPV.

In Patient 2, there was a challenging combination of peripheral signs and concern for a central cause. Central signs were confirmed after consultation with the acute vertigo specialist and the use of video goggles. This prompted a brain MRI, which revealed an acute infarction, accounting for the patient's initial symptoms at first presentation 10 days prior.

The Dix-Hallpike test (DHT) is underutilized even in patients whose symptoms are consistent with BPPV. Patient 4 reported to the specialist that his symptoms were intermittent and exacerbated by changes in head position. The specialist found no focal neurological deficits and performed the DHT, which showed clear posterior canal BPPV. This patient summary underscores the importance of applying the DHT to the appropriate patient population and avoiding delay in the diagnosis and treatment of BPPV.

All four patients could have avoided a CT scan if they had been referred to the acute vertigo service prior to imaging. It is recognized that CT scans have a low sensitivity (7–16%) for identifying acute ischemic stroke in the posterior fossa (Saber Tehrani et al., 2018). This is reflected in the recent GRACE-3 guidelines, which suggest not using CT in the management of patients presenting with acute vertigo and dizziness (Edlow et al., 2023). Unaware of these limitations, inappropriate scans such as CT may give a false sense of reassurance and could be interpreted as a normal scan, ruling out an acute problem.

### 3.2. The role of acute vertigo services in the hyperacute setting

**Figure 1:**
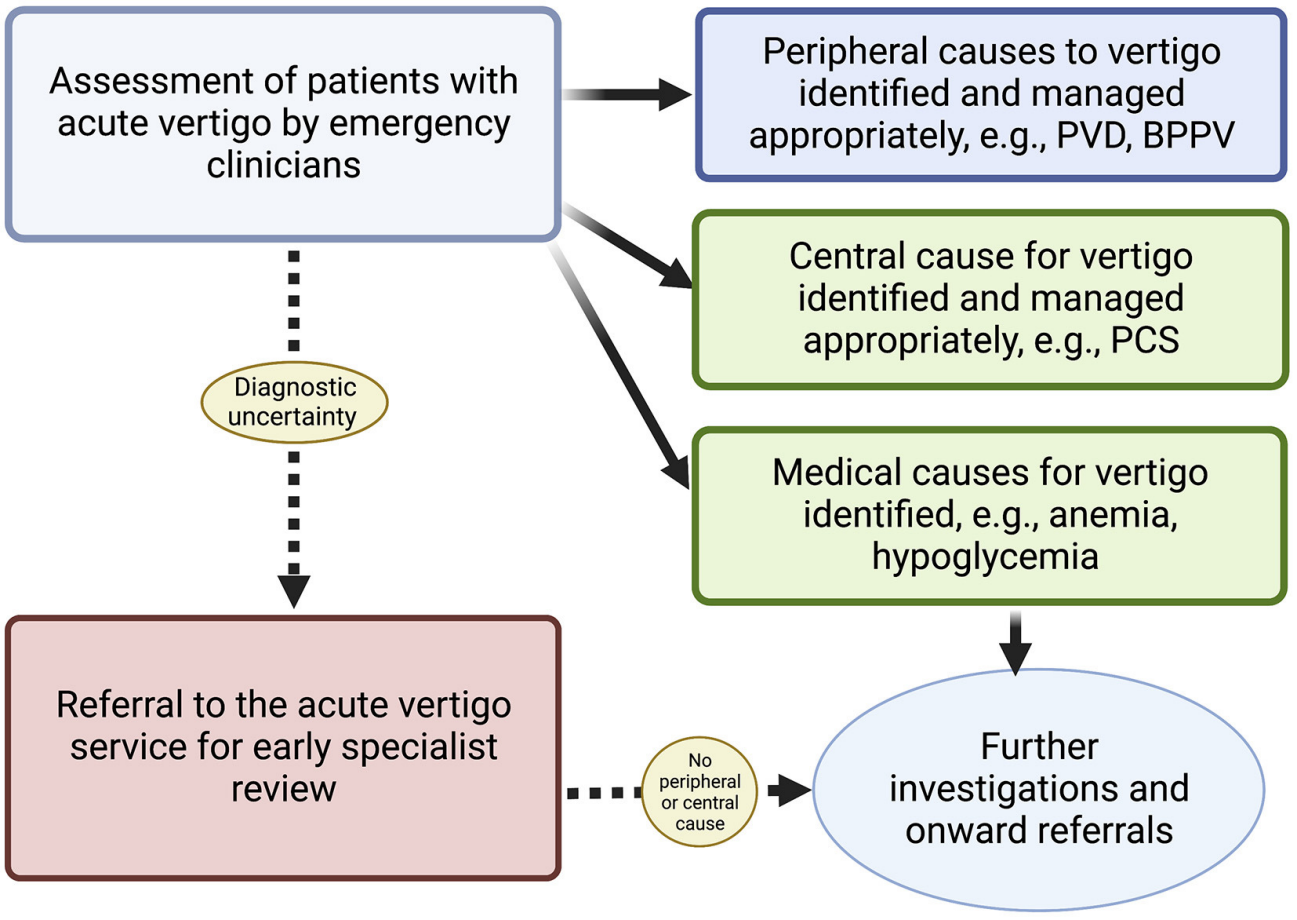
Suggested pathway for patients with dizziness in the hyperacute setting.

Hyperacute vertigo input provides a rapid specialist assessment within the crucial 24-h period from symptom onset when critical management decisions need to be made. Specialist involvement streamlines this process, avoids unnecessary tests such as CT scans, and assists non-specialist teams in discharging patients with appropriate follow-up. Close collaboration between specialist and non-specialist acute vertigo teams is crucial to properly selecting patients for review and triaging patients with non-neurological causes for their symptoms. Collaboration also enables shared learning between teams and helps refine the patient pathway (Figure 1).

Hyperacute specialist input is helpful in identifying the rare patient with a central cause of their acute vestibular syndrome. In some patients, signs such as peripheral nystagmus may develop into central nystagmus over minutes to hours despite normal imaging (Kaski et al., 2023). The lack of specialist input in such cases likely accounts for the high rates (~30%) of stroke misdiagnosis in patients presenting with acute vertigo (Saber Tehrani et al., 2018).

These patient summaries reflect that input from hyperacute vertigo specialists helps make diagnoses and advance patient care, as others have also suggested (Getz et al., 2023; Tarnutzer et al., 2023). However, there is a significant mismatch between the number of neuro-otologists (and other acute vertigo specialists) and the prevalence of dizziness in the acute setting. It is important that clinicians who see many of these patients, such as emergency physicians, are trained in the assessment of acute vertigo, understand the need to screen for neurological features (e.g., diplopia, dysarthria, truncal ataxia), and use diagnostic tools such as the HINTS-plus assessment appropriately (i.e., only in patients with AVS with nystagmus). Having an acute vertigo service embedded within these teams enables educational opportunities to help develop these skills and equip emergency clinicians to make decisions. This may include training in the execution of the Dix-Hallpike maneuver to identify pearls and pitfalls in its performance and interpretation (Tahtis et al., 2021).

Access to acute vertigo specialist input helps provide a specialist opinion when patients have been seen by neurologists or stroke physicians who have adequate experience in assessing patients with acute dizziness. This is highlighted for patients 1 and 3, who were referred from the HASU for non-stroke diagnoses after review by a stroke specialist.

### 3.3. Challenges in providing a hyperacute vertigo service

Effectively running an acute vertigo service requires sufficient staff to consistently provide timely patient assessments and investigations in a time-pressured setting. Providing an out-of-hours service is also challenging, as it requires a significant number of clinicians to offer a 24-h on-call service. A proposed starting point would be a 9 am to 5 pm service, which would enable patients who present overnight to be reviewed in the morning, or for specialists to be available in the early hours of the day. If such an approach is successful, funding streams could be sought to help expand the service further.

A major challenge to providing a hyperacute vertigo service is the shortage of neuro-otologists (and other acute vertigo specialists) in the UK and worldwide, with neuro-otologists comprising <2% of the consultant workforce in the UK (Nitkunan et al., 2020). Improving this situation will take time, and vestibular neurology needs to be embedded in the neurology training curriculum to help develop the specialists of the future.

A multitude of skills are required to deal with acute neurological presentations, namely general medical, cardiovascular, neurovascular, and neuro-otological expertise. A model that can efficiently accommodate all of these care streams has the potential to have a transformative impact. By addressing the lack of formal training in the current validated algorithms (e.g., HINTS-plus, STANDING, etc.) and teaching them how to screen for central neurological features and accurately record initial findings, frontline clinicians in emergency settings can be equipped to make prompt decisions, even in hospitals that have minimal access to specialists and technology such as video oculography (Dmitriew et al., 2021).

## Data availability statement

The original contributions presented in the study are included in the article/supplementary material, further inquiries can be directed to the corresponding authors.

## Ethics statement

Written informed consent was obtained from the individual(s) for the publication of any potentially identifiable images or data included in this article.

## Author contributions

WB: Formal analysis, Methodology, Project administration, Writing—original draft, Writing—review and editing. SH: Conceptualization, Data curation, Methodology, Supervision, Visualization, Writing—review and editing. MB: Resources, Writing—review and editing. AA-M: Resources, Writing—review and editing. AC: Methodology, Resources, Writing—review and editing. RS: Methodology, Supervision, Writing—review and editing. NK: Conceptualization, Methodology, Project administration, Resources, Visualization, Writing—review and editing. DK: Conceptualization, Data curation, Methodology, Supervision, Validation, Visualization, Writing—original draft, Writing—review and editing.
